# Drug Discovery and Development of miRNA-Based Nucleotide Drugs for Gastrointestinal Cancer

**DOI:** 10.3390/biomedicines11082235

**Published:** 2023-08-09

**Authors:** Hiromichi Sato, Tomoaki Hara, Sikun Meng, Yoshiko Tsuji, Yasuko Arao, Kazuki Sasaki, Norikatsu Miyoshi, Shogo Kobayashi, Yuichiro Doki, Hidetoshi Eguchi, Hideshi Ishii

**Affiliations:** 1 Center of Medical Innovation and Translational Research, Department of Medical Data Science, Osaka University Graduate School of Medicine, Yamadaoka 2-2, Suita 565-0871, Osaka, Japan; hiromichi7286@gmail.com (H.S.);; 2 Department of Gastrointestinal Surgery, Osaka University Graduate School of Medicine, Yamadaoka 2-2, Suita 565-0871, Osaka, Japan

**Keywords:** epigenetic reprogramming, induced pluripotent stem cells, miRNA-modified nucleotide drugs, gastrointestinal cancers

## Abstract

Short non-coding RNAs, miRNAs, play roles in the control of cell growth and differentiation in cancer. Reportedly, the introduction of miRNAs could reduce the biologically malignant behavior of cancer cells, suggesting a possible use as therapeutic reagents. Given that the forced expression of several miRNAs, including miR-302, results in the cellular reprograming of human and mouse cells, which is similar to the effects of the transcription factors Oct4, Sox2, Klf4, and c-Myc, this suggests that the selective introduction of several miRNAs will be able to achieve anti-cancer effects at the epigenetic and metabolic levels. In this review article, we bring together the recent advances made in studies of microRNA-based therapeutic approaches to therapy-resistant cancers, especially in gastrointestinal organs.

## 1. Introduction

In mammalian embryos, epigenetic reprogramming of the genome occurs in two major cycles, preimplantation development and germ cell development [1]. Since sequences that escape reprogramming may be involved in epigenetic inheritance, it is important to examine whether reprogramming is involved in stem cell differentiation and in germ cells and early embryos [1]. Epigenetic modifications of DNA, such as methylation, are important for genomic function during development [2]. In adults, DNA methylation is of central importance for genomic imprinting and other epigenetic regulations of gene expression, and methylation patterns are largely maintained in somatic lineages during development [3]. Mammalian genomes undergo major reprogramming of methylation patterns in germ cells and early embryos [3]. Subsequently, reprogramming occurs in mammals and flowering plants, and the similarities and differences reveal strategies of development and reproduction [4].

Blastocysts cloned from melanoma nuclei induce embryonic stem (ES) cells with the potential to differentiate into multiple cell types in vivo, including melanocytes, lymphocytes, and fibroblasts, and it has been argued that the malignant phenotype of certain cancer cells can be reversed to a pluripotent state despite the presence of irreversible genetic alterations [5]. This has led to an understanding of the relationship between reversal to the pluripotent state and changes in the malignant phenotype of cancer cells.

**Figure 1:**
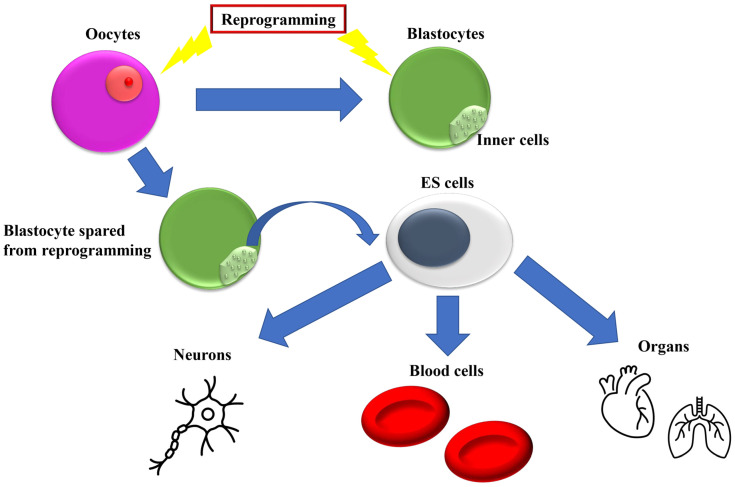
Blastocyst-derived embryonic stem (ES) cell progress developmental program, achieved by reprogramming mechanism. In mammalian embryos, epigenetic reprogramming of the genome occurs in two major cycles: preimplantation development and germline development. Blastocysts that escape reprogramming can transform into ES cells and differentiate into various cell types.

Other studies compared the transcriptional profiles of cloned mouse blastocyst-derived ES cells (nuclear transplantation (NT)-ES cells) and fertilization-derived ES cell lines, reporting that cloned and fertilized mouse blastocyst-derived ES cell lines are indistinguishable in their transcriptional profiles and are consistent with normal developmental potential [6]. In contrast to clonal embryo–fetal development, during the process of NT-ES cell induction, immortal cells that have erased the “epigenetic memory” of the donor nucleus are strictly selected, resulting in functional equivalence (Figure 1) [6].

## 2. Introduction of Transcription Factors for Reprogramming

Considering the ethical issues of using fertilized eggs for the establishment and production of ES cells and the immunological compatibility that arises in the case of unrelated donors, Takahashi and Yamanaka reported that complete initiation could be achieved by introducing biological factors, such as Oct4, Sox2, Klf4, and c-Myc, into mouse and human fibroblasts [7,8]. Furthermore, they reported that they made a breakthrough by introducing complete initiation [7,8].

Previous studies showed that these four factors are not necessarily essential: neural stem cells from adult mice express higher levels of Sox2 and c-Myc intrinsically than ES cells, and exogenous Oct4 along with Klf4 or c-Myc is sufficient to generate induced pluripotent stem cells (iPSCs) from neural stem cells, suggesting that the number of initiating factors can be reduced [9]. Sall4 is a zinc finger transcription factor and is expressed very early in embryogenesis along with Oct4 and Nanog, two well-known pluripotency regulators [10]. When Sall4 expression is reduced, the expression levels of four proteins that function to initialize somatic cells into induced pluripotent cells, Oct4, Sox2, c-Myc, and Klf4, were reduced, which suggests that Sall4 plays a diverse role in early embryogenesis, integrating transcriptional and epigenetic regulation, and regulating stem cell pluripotency [10]. The orphan nuclear receptor Nr5a2 (also known as Lrh-1) has been shown to substitute for Oct4 in the induction of iPSCs from mouse somatic cells to enhance initiation efficiency [11]. Genome-wide positional analysis revealed that Nr5a2 shares many common gene targets with Sox2 and Klf4, suggesting that this trio of transcription factors cooperate to mediate initiation [11]. Furthermore, we found that Nr5a2 functions in part by activating Nanog [11]. Subsequent studies showed that NKX3-1, a prostate-specific tumor suppressor, plays an important role in iPSC initiation [12], and the NCoR/SMRT corepressor binds to the pluripotency locus and creates a barrier to initiation by the four Yamanaka factors (Oct4, Sox2, Klf4, and c-Myc), resulting in a significant increase in the efficiency and speed of initiation by suppressing NCoR/SMRT [13].

However, it became clear that iPSCs alone could not efficiently generate chimeric mice because the promoters of immature genes, such as Oct4, are heavily methylated [14]. Although it is possible to augment the initiation process with chemicals, the problems of low initiation efficiency and residual vector sequences in many protocols remain unresolved, and it is necessary to establish reprogramming with miRNAs with improved safety and efficiency [15]. Hence, some reports have devised ways to increase the generation of induced pluripotent cancer (iPC) cells through hypoxia or TP53 deficiency [16].

## 3. Resolution of Initiation Efficiency by Forced Expression of miRNAs

Several studies reported that human and mouse somatic cells could be reprogrammed into iPSCs by the forced expression of miRNAs [15,17]. Induction of the expression of immature state-related proteins, such as Nanog, Ssea4, Tra-1–60, and Tra-1–80, requires retroviral iPSC gene transfer. Pluripotency through iPSC transfection was subsequently confirmed at other institutions, and the technology confers greater sensitivity to chemotherapeutic agents [17]. Furthermore, long-term cultured iPC cells from cholangiocarcinoma HuCC-T1 cells showed resistance to 5-fluorouracil and exhibited high tumorigenic potential due to endogenous c-Myc [18]. Using the potent transcription activation domain of the Myc protein, a human Oct4 fusion protein was created, which significantly improved the expression of the targeted genes and increased the initiation efficiency compared to Oct4 alone [19].

**Figure 2:**
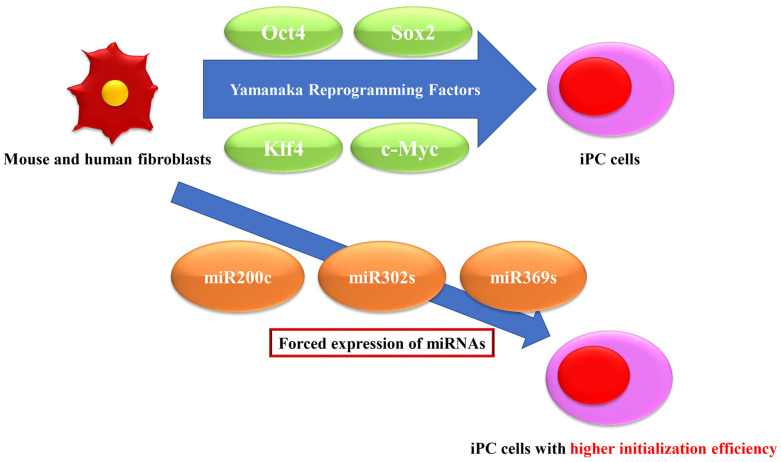
Improvement of initiation efficiency by transfection of miRNAs. Although the Yamanaka reprogramming factors successfully generated induced pluripotent cancer (iPC) cells from mouse and human fibroblasts, the initiation efficiency was low, and transfection of miRNAs resulted in the introduction of iPC cells with higher initiation efficiency.

Conversely, Miyoshi et al. showed that it is possible to initiate mouse and human cells to pluripotency by direct introduction of mature double-stranded miRNAs using a combination of mir-200c plus mir-302s and mir-369s [15]. In that report, Nanog was significantly dimethylated in clones to which miRNAs were added [15]. Miyazaki et al. found that direct transfection of miRNAs (miR-200c, -302s, and -369s) can reprogram mouse and human cells to become pluripotent [20]. Metastatic reprogramming using human colon cancer cells transplanted into mice altered Nanog methylation and stably increased Nanog and NANOGP8 [21]. The use of mir-302/367 alone or combined with Yamanaka reprogramming factors has made it possible to establish iPSCs with high efficiency (Figure 2) [22]. MiR-302 promotes pluripotency by inhibiting NR2F2 through a feedback loop [23]. MiR-19a/b enhances iPSC induction efficiency by targeting and inhibiting the tumor suppressor phosphatase and tensin homolog (PTEN), which is associated with oncogenic signaling in human malignancies [24]. MiR-132 and miR-212 impair endogenous epigenetics by suppressing two important epigenetic regulators, histone acetyltransferase p300 and H3K4 demethylase Jarid1a (KDM5a) [25].

In past reports, miRNAs have also received attention as antiangiogenic agents and as angiomiRs that can contribute to the treatment of various diseases such as diabetic retinopathy, rheumatoid arthritis, and cancer, which are associated with unbalanced angiogenesis [26]. Because some patients with metastatic renal cell carcinoma treated with tyrosine kinase inhibitors (TKIs) are resistant to angiogenesis inhibitors, miRNAs that predict disease progression under TKI treatment were identified (miR-1307-3p, miR-155-5p, miR-221-3p) and a new set of related markers is also being attempted to provide a new set of markers [27]. It has been shown that miRNA therapeutics for tumors are based on targeting or mimicking miRNAs involved in cancer initiation, progression, epithelial–mesenchymal transition, and metastasis as well as this angiogenesis, that miRNA replacement therapy enhances the anti-tumor effect of anti-tumor drugs, and that the anti-miRNA and miRNA replacement strategy combination may provide superior results in terms of anti-tumor efficacy. This is one of the areas that has attracted much attention in recent years. With miRNA profiles being proposed as a diagnostic tool and promising therapy in the breast cancer field, extracellular miRNAs may become a new, easily accessible, affordable, and non-invasive tool for breast cancer patients [28], and miRNAs have been shown to be useful in the treatment of triple-negative breast cancer in the light of their influence on tumor development, progression, invasion, and metastasis [29].

The development of new and superior therapies for cancers that are resistant to standard therapies is urgently needed, and the involvement of miRNAs, especially in therapy-resistant gastrointestinal cancers, will be discussed in the next chapter [30].

## 4. Application to Refractory Gastrointestinal Cancer

### 4.1. Liver

The miR-302-mediated iPSC technology by Koga et al. caused H3K4 methylation and c-Myc suppression through AOF2 downregulation, reprogramming hepatocellular carcinoma (HCC) cells, and improving drug sensitivity [31]. MiR-451 functions as a metastasis suppressor of HCC cells by the direct suppression of IKK-β and partially targeting c-Myc, which may be a novel prognostic biomarker for patients with HCC [32,33]. As a tumor suppressor, miR-214 inhibits HCC tumorigenesis by suppressing β-catenin [34]. Additionally, miR-375 targets AEG-1 and inhibits the growth of HCC [35]. MiR-105 also functions as a tumor suppressor against HCC by inhibiting the PI3K/AKT signaling pathway [36]. The downregulation of TLR2, NF-κB, MMP-2, and MMP-9 expression by expressing miR-143 significantly inhibits HCC growth and invasion and promotes apoptosis [37]. MiR-15b-5p overexpression suppresses Rab1A and inhibits HCC cell growth, suggesting that it acts as a tumor suppressor gene in HCC [38]. Transfection with miR-125a/b inhibits cell proliferation by targeting CD90, a stem cell marker of HCC stem cells [39]. However, miR-423 contributes to growth promotion in hepatocarcinogenesis via the suppression of the expression of the tumor suppressor p21Cip1/Waf1 [40]. Hepatitis C virus infection promotes hepatocyte proliferation and tumorigenesis through the high expression of miR-155 and the activation of Wnt signaling [41]. MiR-362-5p activates the NF-κB signaling pathway via cylindromatosis (CYLD) to induce contributions to tumor growth [42]. MiR-342 regulates the NF-κB pathway, affects HCC cell proliferation, and partially restores hepatocyte proliferation by overexpressing Ikk-γ, TAB2, and TAB3 [43]. Patients with HCC with high miR-139 expression have longer overall survival than those with low expression, and karyopherin alpha 2, a direct target of miR-139, is associated with poor patient prognosis [44].

### 4.2. Colon and Rectum

The introduction of mature miRNAs (miR-302a-d, 369-3p and 5p, and miR-200c) into colon cancer cells suppressed c-Myc expression [45]. Ogawa et al. showed that the introduction of miR-302s and miR-369s induced cellular initiation and suppressed the malignant phenotype of human colon cancer [46]. The expression of the calcium-sensing receptor (CaSR) in human colonic crypt epithelium is associated with cell differentiation, its absence is associated with undifferentiated and invasive colon cancer, and CaSR-deficient cells show an increased expression of the cancer stem cell markers CD133, CD44, Nanog, and epithelial mesenchymal transition molecules, the transcription factors N-cadherin, beta catenin, vimentin, fibronectin, Snail1, Snail2, Twist, FOXC2, and oncogenic miR21, miR135a, and miR135b [47]. On the other hand, the tumor suppressors E-cahelin and miR145 were shown to be markedly downregulated in CaSR-deficient cells [47]. LincRNA-p21 inhibits the activity of β-catenin signaling and significantly suppresses the self-renewal and tumorigenic potential of colon cancer stem cells [48]. Wnt10b is a downstream target of miR-148a, and the overexpression of miR-148a suppresses Wnt10b expression and β-catenin signaling activity, at least partially increasing stem cell marker expression and chemotherapy sensitivity, and suppresses cell invasion and migration [49]. Tumor-derived exosome miR-934 promotes colorectal cancer liver metastasis by regulating cross-talk between colon cancer cells and tumor-associated macrophages [50].

In the area of colorectal cancer, various clinical trials have been conducted, and analysis of miRNA expression not only in patients’ serum [51] but also in colon cells isolated from feces [52] has shown high expression of the aforementioned miR21, which is attracting attention as a screening test in the future.

### 4.3. Pancreas

The expression of miR-744 enhances the Wnt/β-catenin signaling pathway, which is commonly enhanced in pancreatic cancer, and augments tumorigenic potential, suggesting that miR-744 may be a novel prognostic biomarker and therapeutic target [53]. Similarly, another report showed that miR-148a inhibits epithelial–mesenchymal transition and the invasion of pancreatic cancer cells by targeting Wnt10b and inhibiting the Wnt/β-catenin signaling pathway [54]. Other potential new therapeutic targets for pancreatic cancer, such as the sex-determining region Y-box2 (Sox2) and the signal transduction and activation of transcription 3 (STAT3), are suppressed by miR-1181 [55]. M2 macrophage-derived exosome miR-501-3p activates the TGF-β signaling pathway and promotes pancreatic ductal adenocarcinoma development by suppressing the tumor suppressor TGFBR3 gene [56].

### 4.4. Esophagus

MiR-942 directly binds to c-Myc and upregulates Wnt/β-catenin signaling activity in esophageal squamous cell carcinoma (ESCC), suggesting that miR-942 is an effective therapeutic target [57]. The tumor suppressor miRNAs, miR-145, miR-133a, and miR-133b, directly regulate FSCN1 to suppress cell proliferation and invasion in ESCC cells [58]. Although miR-196a regulates RAS-related protein (RAP1A) expression, single-nucleotide polymorphism rs6573 inhibits the binding of miR-196a to the RAP1A 3‘UTR and increases the constitutive expression of RAP1A, which is overexpressed in the majority of ESCCs [59]. Conversely, other reports showed that a high expression of miR-142-3p results in ESCCs with poor prognosis and could be a prognostic biomarker, which may be caused by regulating transcription, cell cycle, and differentiation. MiR-142-3p is also involved in cell migration and adhesion, transcriptional activation, and apoptosis [60]. MiR-138 functions in a tumor-suppressive manner for ESCC. MiR-138 suppression promotes K63-bound polyubiquitination of NF-κB signaling intermediates, TRAF2 and RIP1, to sustain NF-κB activation and in lipid rafts, which are small (10–200 nm), liquid-ordered regions with cholesterols and sphingoglycolipids, including FLOT1, FLOT2, and caveolin-1. Its downregulation also induces lipid raft formation via the upregulation of multiple components [61]. MiR-183 directly targets PDCD4, and the overexpression of miR-183 promotes ESCC cell proliferation and invasion [62,63].

### 4.5. Stomach

In gastric cancer (GC), tumor macrophage-derived miR-21 trafficking confers resistance to cisplatin (CDDP) and is a novel therapeutic strategy [64]. Many other miRNAs have been reported to be involved in resistance to cisplatin. MiR-588 targets CYLD in GC cells and promotes the growth of CDDP-resistant GC cells by depleting CYLD [65]. The subcutaneous injection of exo-anti-214, an exosome loaded with miR-214, downregulates miR-214 in tumors, reverses chemotherapy resistance, and inhibits tumor growth in vivo [66]. Exosome-secreted miR-107 enhances sensitivity to CDDP via HMGA2/mTOR/P-gp [67]. HOX transcript antisense RNA (HOTAIR) binds directly to miR-34a, and the knockdown of HOTAIR enhances the inhibitory effect of CDDP on tumor growth in vivo [68]. Elevated miR-500a-3p in exosomes increases CDDP resistance and poor progression-free progression by targeting FBXW7 [69]. Turning to doxorubicin, exosomal miR-501 induces resistance to doxorubicin by the downregulation of cell death inducer (BLID), the inactivation of caspase-9/3, and the phosphorylation of Akt [70]. MiR-487a derived from M2 macrophages regulates GC progression and promotes the proliferation and tumorigenesis of GC by targeting TIA1 [71]. Exo-miR-519a-3p has also been reported to play an important role in the cross-talk between primary GC tumor cells and intrahepatic macrophages by targeting DUSP2 and activating the MAPK/ERK pathway [72]. In another report, the macrophage-derived exosome miR-223 promotes GC cell metastasis via the PTEN-PI3K/AKT pathway [73]. Exosomal miR-106a has also been reported to promote tumor growth by targeting Smad7 and can contribute to GC peritoneal metastasis (Table 1) [74]. The results of these studies suggest that the appropriate introduction of small functional ribonucleic acid opens new therapeutic avenues against human malignancies [75].

## 5. Future Prospects

As mentioned above, miRNA-based nucleic acid drugs are becoming known to be a simple and effective treatment for drug-resistant cancers, and the development of next-generation therapeutics may make great progress not only in the field of cancer but also in the field of irritable bowel syndrome [76], cardiovascular diseases [77], and obesity control [78]. In addition to the carcinomas discussed in Section 4, a clinical trial of small-bowel neuroendocrine tumors found serum miR-125b-5p and miR-362-5p to be useful in the detection of residual disease/recurrent disease [79]. Considering the lncRNA-miRNA-mRNA network in response to lactobacillus supplementation, a strong correlation between miR and genetic variants (e.g., miR-133b and IGF1 gene) was also explored to provide a novel monitoring and therapeutic approach, and a randomized controlled trials are under way [80]. Recently, it has been shown that the computational prediction of functional microRNA-mRNA interactions may lead to a better understanding of functional interactions and pave the way for the discovery of disease markers and the design of miRNA-based drugs [81]. It is clear that this is an area that will continue to be studied not only in gastrointestinal cancer but also in many other fields. However, one of the most critical aspects of using miRNAs is the current lack of safe, reliable, and robust methods to selectively target organs and tissues, and thus, the development of cell-specific delivery vehicles is an essential step for the clinical application of miRNA-based therapeutics for cancer management. Therefore, the development of cell-specific delivery methods will be an essential step in the clinical application of miRNA-based therapeutics for cancer management, and will be eagerly awaited.

## 6. Conclusions

Cancer cells can be initiated into iPC cells by specific transcription factors and miRNAs, similar to the initiation of normal fibroblasts into iPSCs. iPC cells can improve sensitivity to conventional chemotherapy [82]. However, the disadvantage of this miRNA-based therapy is that it is not suitable for systemic therapy due to the presence of RNase in the blood. While miRNAs have been considered efficacious for use in anticancer drugs in research on colorectal cancer, hemodynamics and delivery have been major issues [83].

**Figure 3:**
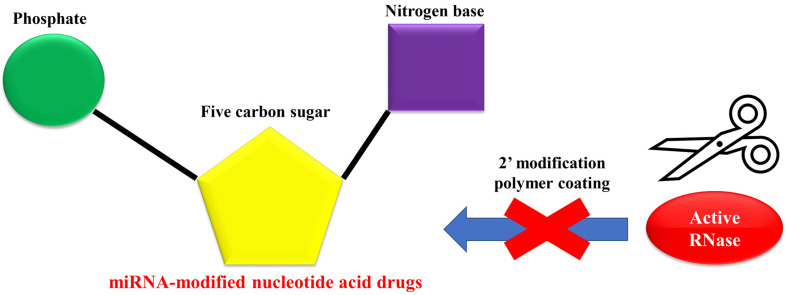
Ensuring the stability of therapeutic drugs by introducing “miRNA-based nucleotide acid drugs”. The problem with miRNA-based therapy is that it is not suitable for systemic treatment due to the presence of RNase in the blood. Therefore, the introduction of “miRNA-based nucleotide drugs” can maintain the stability of therapeutic agents.

To address such issues, self-assembling nanocomplexes formed by miRNA mimetics and multifunctional peptide conjugates were used to deliver tumor suppressive miR-34a and the multifunctional peptide conjugate FA-R9-FPcas3 to HeLa cells at high rates [84]. Experimentally, the direct binding of miRNAs to aptamers for transmembrane receptors may provide an efficient delivery tool [85]. Extracellular vesicles (EVs), including exosomes, microvesicles, and apoptotic bodies, are nano-sized membrane vesicles derived from most cell types and are important mediators of intercellular signal transduction, and therefore, in the treatment of various diseases, not only miRNAs but also siRNAs, proteins, small molecule drugs, nanoparticles, and CRISPR/Cas9 have been reported to be useful in drug delivery systems [86]. Currently, attempts are being made to develop modified miRNAs that maximize antitumor effects by applying chemical modifications to the guide and passenger strands and measuring luciferase activity. Therefore, this “miRNA-based nucleotide medicine” has recently been used to improve the stability and efficacy of miRNA-based therapy, with the aim of applying it to real-world clinical practice (Figure 3).

## Figures and Tables

**Table 1:** Relationships between various gastrointestinal cancers and miRNAs reported so far.

Cancer Type	miRNA Type	Proliferative or Inhibitory Action against Cancer	Molecular Mechanism of Action Sequence
Liver	miR-302	Inhibitory	H3K4 methylation and c-Myc suppression through AOF2 downregulation and reprogramming of hepatocellular carcinoma (HCC) cells [31]
	miR-451	Inhibitory	Direct suppression of IKK-β and partially targeting c-Myc [32,33]
	miR-214	Inhibitory	Suppression of β-catenin [34]
	miR-375	Inhibitory	Targets AEG-1 and inhibits the growth of HCC [35]
	miR-105	Inhibitory	Inhibits the PI3K/AKT signaling pathway [36]
	miR-143	Inhibitory	Downregulates TLR2, NF-κB, MMP-2, and MMP-9 expression, inhibits HCC growth and invasion, and promotes apoptosis [37]
	miR-15b-5p	Inhibitory	Suppresses Rab1A and inhibits HCC cell growth [38]
	miR-125a/b	Inhibitory	Inhibits cell proliferation by targeting CD90, a stem cell marker of HCC stem cells [39]
	miR-423	Proliferative	Suppression of expression of the tumor suppressor p21Cip1/Waf1 [40]
	miR-155	Proliferative	Hepatitis C virus infection promotes hepatocyte proliferation and tumorigenesis and simultaneously activates Wnt signaling [41]
	miR-362-5p	Proliferative	Activates the NF-κB signaling pathway via cylindromatosis (CYLD) to induce contributions to tumor growth [42]
	miR-342	Proliferative	Regulates the NF-κB pathway and overexpresses Ikk-γ, TAB2, and TAB3 [43]
	miR-139	Inhibitory	Regulates karyopherin alpha 2 [44]
Colon and rectum	miR-302a-d, 369-3p and 5p, and miR-200c	Inhibitory	Suppresses c-Myc expression [45]
	miR-302s and miR-369s	Inhibitory	Induces cellular initiation and suppresses the malignant phenotype of human colon cancer [46]
	miR21, miR135a, and miR135b	Proliferative	Suppresses calcium-sensing receptor (CaSR) in human colonic crypt epithelium, which is associated with cell differentiation [47]
	miR145	Inhibitory	Upregulates CaSR cells [48]
	miR-148a	Inhibitory	Suppresses cell invasion and migration by targeting WNT10b and β-catenin signaling activity [49]
	miR-934	Proliferative	Promotes colorectal cancer liver metastasis by regulating cross-talk between colon cancer cells and tumor-associated macrophages [50]
Pancreas	miR-744	Proliferative	Enhances the Wnt/β-catenin signaling pathway, which is commonly enhanced in pancreatic cancer, and augments the tumorigenic potential [53]
	miR-148a	Inhibitory	Inhibits epithelial–mesenchymal transition and invasion of pancreatic cancer cells by targeting Wnt10b and inhibiting the Wnt/β-catenin signaling pathway [54]
	miR-1181	Inhibitory	Suppresses sex-determining region Y-box2 (SOX2) and signal transduction and activation of transcription 3 (STAT3) [55]
	miR-501-3p	Proliferative	Activates the TGF-β signaling pathway and promotes pancreatic ductal adenocarcinoma development by suppressing the tumor suppressor TGFBR3 gene [56]
Esophagus	miR-942	Proliferative	Directly binds to c-Myc and upregulates Wnt/β-catenin signaling activity [57]
	miR-145, miR-133a, and miR-133b	Inhibitory	Directly regulates FSCN1 to suppress cell proliferation and cell invasion in esophageal squamous cell carcinoma (ESCC) cells [58]
	miR-196a	Inhibitory	Regulates RAS-related protein (RAP1A) expression Single-nucleotide polymorphism rs6573 inhibits the binding of miR-196a to the RAP1A 3′UTR and increases the constitutive expression of RAP1A, which is overexpressed in the majority of ESCCs [59]
	miR-142-3p	Inhibitory	Regulates transcription, cell cycle, and differentiation and is also involved in cell migration and adhesion, transcriptional activation, and apoptosis [60]
	miR-138	Inhibitory	Its suppression promotes K63-bound polyubiquitination of NF-κB signaling intermediates, TRAF2 and RIP1, to sustain NF-κB activation and in lipid rafts, including FLOT1, FLOT2, and caveolin-1, and induces lipid raft formation via upregulation of multiple components [61]
	miR-183	Proliferative	Directly targets PDCD4, and overexpression of miR-183 promotes ESCC cell proliferation and invasion [62,63]
Stomach	miR-21	Proliferative	Confers resistance to cisplatin (CDDP) [64]
	miR-588	Proliferative	Targets CYLD in gastric cancer (GC) cells and promotes the growth of CDDP-resistant GC cells by depleting CYLD [65]
	miR-214	Proliferative	Downregulated miR-214 in tumors, reverses chemotherapy resistance, and inhibits tumor growth in vivo [66]
	miR-107	Proliferative	Enhances sensitivity to CDDP via HMGA2/mTOR/P-gp [67]
	miR-34a	Proliferative	HOX transcript antisense RNA (HOTAIR) binds directly to miR-34a, and the knockdown of HOTAIR enhances the inhibitory effect of CDDP on tumor growth in vivo [68]
	miR-500a-3p	Proliferative	Increases CDDP resistance and poor progression-free progression by targeting FBXW7 [69]
	miR-501	Proliferative	Induces resistance to doxorubicin by downregulation of cell death inducer (BLID), inactivation of caspase-9/3, and phosphorylation of Akt [70]
	miR-487a	Inhibitory	Regulates GC progression and promotes proliferation and tumorigenesis of GC by targeting TIA1 [71]
	519a-3p	Proliferative	Plays an important role in the cross-talk between primary GC tumor cells and intrahepatic macrophages by targeting DUSP2 and activating the MAPK/ERK pathway [72]
	miR-223	Proliferative	Promotes GC cell metastasis via the PTEN-PI3K/AKT pathway [73]
	miR-106a	Proliferative	Promotes tumor growth by targeting Smad7 and can contribute to GC peritoneal metastasis [74]

## Data Availability

The data that support the findings of this study are available from the corresponding author upon reasonable request.
